# Lipoprotein Metabolism in Hematological Malignancies: A Role in Shaping the Tumor Cell Microenvironment?

**DOI:** 10.3390/metabo16020145

**Published:** 2026-02-20

**Authors:** Manal Sellam, Mélanie Lambert, Nadine Varin-Blank, Kevin Saitoski

**Affiliations:** 1 Institut Universitaire et Technologique, Université Sorbonne Paris Nord, 93017 Bobigny, France; manal.sellam1@edu.sorbonne-paris-nord.fr (M.S.); melanie.lambert@inserm.fr (M.L.); kevin.saitoski@inserm.fr (K.S.); 2 Institut National de la Santé et de la Recherche Médicale (INSERM), 93017 Bobigny, France

**Keywords:** lipoprotein metabolism, leukemia, lymphoma, tumor progression

## Abstract

The tumor microenvironment (TME) plays a key role in driving tumor progression, metastasis, and resistance to therapy. The TME is a highly variable ecosystem composed of both cancer and surrounding normal cells, immune survey cells and the extracellular matrix, also composed of signaling molecules that mediate interactions between them. Blood cancer cells pose a unique challenge because of their circulation and widespread distribution along with their capacity to invade various niches, interacting with a wide range of host cells such as fibroblasts, immune cells, endothelial cells, and adipocytes. Metabolism reprogramming in this tumor context, notably referring to elevated cholesterol and fatty acid metabolism, emerges as a crucial event in shaping an immune-suppressive microenvironment that promotes tumor progression. Cholesterol and fatty acids are supplied by both de novo biosynthesis and exogenous uptake from lipoproteins. Lipoproteins are pseudo-micellar structures, designed to transport essential water-insoluble metabolites, including triacylglycerols and cholesterol, in the plasma, lymph, and interstitial fluids. A number of studies have reported abnormal circulating lipoprotein levels in leukemic patients and have suggested that lipoproteins are key for cancer cells to thrive. However, the role of lipoprotein metabolism in cancer cells in the context of the TME is still incompletely discussed so far. The aim of this review is to consider the importance of lipoprotein metabolism in shaping the tumor microenvironment in the context of hematological malignancies.

## 1. Introduction

Hematological malignancies comprise a heterogeneous group of myeloid and lymphoid neoplasms caused by the disruption of normal hematopoiesis. They are classified into three main subtypes: leukemia, myeloma, and lymphoma [1]. Non-specific chemotherapy and radiotherapy were the bulk of traditional treatments for hematologic malignancies, which, while effective in eradicating malignant cells, often resulted in severe toxicity and relapse due to damage to normal hematopoietic tissues. In recent years, there has been a significant evolution in the treatment of hematologic malignancies due to a better understanding of the molecular and immune mechanisms underlying these diseases [2]. Investigating the underlying mechanisms and identifying new prognosis markers and novel therapeutic targets are therefore crucial to improve the management of these diseases.

Cancer-specific metabolic alterations have emerged as therapeutically exploitable vulnerabilities across multiple malignancies. Metabolic reprogramming is considered a hallmark of cancer, as tumor cells rely on altered pathways to support energy production and biomass synthesis. Early metabolic studies in hematological malignancies primarily focused on mitochondrial function and cellular respiration. Although some leukemic cells, like many cancer cells, exhibit high glycolytic activity even under aerobic conditions (Warburg effect), they also display a strong dependence on mitochondrial metabolism and oxidative phosphorylation for survival. Because mitochondrial metabolism is essential for all proliferating cells, the pharmacological targeting of this pathway remains challenging. Current approaches have shown either limited clinical efficacy or unacceptable systemic toxicity. Consequently, attention has shifted toward alternative metabolic pathways.

Lipids are a group of organic molecules, insoluble in water but soluble in organic solvents. Together with proteins and nucleic acids, they are essential components of cell membranes and building blocks constituting cells [3]. Besides this structural role, lipids are used by cells in energy storage and metabolism and have crucial roles as signaling molecules for many cellular activities [4]. An increasing body of evidence identifies lipid metabolism as a central and recurrently dysregulated pathway in hematological malignancies, including leukemia and lymphoma. Rather than representing a passive metabolic consequence of malignant transformation, alterations in lipid handling actively sustain tumor cell survival, proliferation, and therapy resistance [5]. Clinically, the metabolic rewiring of lipid pathways is consistently associated with adverse prognosis in leukemic patients, underscoring its functional relevance in disease aggressiveness and outcome [6]. Large-scale genomic analyses further highlight the prominence of lipid metabolic alterations in blood cancers. By interrogating genomic data from 10,528 tumors of 32 different cancers, it was observed that genes involved in lipid metabolism were the most frequently altered metabolic category in hematological malignancies, with particularly high enrichment in diffuse large B-cell lymphoma (DLBCL) [7]. These findings position lipid metabolism as a defined vulnerability of hematological cancers.

Among lipid pathways, fatty acid (FA) metabolism has emerged as a critical determinant of malignant fitness. Enhanced FA uptake, synthesis, and oxidation fuel rapid cell growth and promote chemoresistance in acute myeloid leukemia (AML) and chronic lymphocytic leukemia (CLL) [8,9,10,11,12]. Notably, unlike many solid tumors that rely predominantly on aerobic glycolysis [13], CLL cells depend on oxidative phosphorylation, supported by increased FA uptake and utilization [14,15]. Similar oncogenic reliance on FA metabolism has been reported in aggressive lymphoma such as Burkitt and Mantle cell lymphoma (MCL) [5,11]. Malignant hematological cells also markedly enhance their capacity to uptake exogenous fatty acids [16]. This is mediated by an increased expression level of lipid transporters and chaperones like cluster of differentiation 36 (CD36), fatty acid transport proteins (FATPs), fatty acid-binding proteins (FABPs), and members of the solute carrier family 27 (SLC27). Among these molecules, CD36 attracts particular attention: its overexpression has been reported in leukemia [17,18], lymphoma [19,20], and multiple myeloma (MM) [21], where it correlates with enhanced metastatic potential and drug resistance [22], indicating that lipid uptake actively contributes to malignant adaptation. Thus, many therapeutic strategies targeting the FA uptake, synthesis, and oxidation of cancer cells are under investigation.

Cholesterol metabolism represents a second, equally critical lipid axis in hematological malignancies. Cholesterol is essential for membrane integrity, signal transduction, and cell proliferation [23]. Its implication in cancer biology dates back more than a century, when Webb first proposed that cholesterol crystallization within living cells could promote malignancy [24]. Subsequent works have established that cancer cells display increased cholesterol uptake and synthesis, supporting tumor initiation and progression [25,26]. Clinical observations dating back to the 1970s consistently reported altered circulating cholesterol levels in patients with leukemia and lymphoma [27,28]. Leukemic cells exhibit a heightened requirement for cholesterol compared with normal hematopoietic cells, achieved through both increased endogenous synthesis and enhanced uptake of exogenous cholesterol [29]. Remarkably, they also possess the unique ability to sequester excess cholesterol in lipid droplets as cholesterol esters [30]. The pharmacological blockade of cholesterol esterification suppresses cell proliferation in CLL, AML, and chronic myeloid leukemia (CML) [31], highlighting the functional importance of intracellular cholesterol stores. Consistently, multiple epidemiological and clinical studies have linked statin treatment, involving cholesterol-lowering drugs, to reduced cancer risk and improved outcomes in hematological malignancies [32]. However, mechanistic interpretation remains complex, as statins also inhibit the mevalonate pathway, which generates essential intermediates required for cell survival beyond cholesterol synthesis itself [33].

Lipoproteins are the principal plasma carriers of lipids, including cholesterol and fatty acids. Aberrant plasma lipoprotein profiles have been consistently documented in patients with leukemia and lymphoma and correlate with disease pathogenesis and progression. Although the underlying mechanisms remain incompletely understood, these alterations likely reflect the coordinated metabolic reprogramming of both malignant cells and the surrounding tumor microenvironment cells, thereby positioning systemic lipid transport as an integral intermediate in hematological cancer biology.

The aim of the review is to (i) highlight the potential role of lipoprotein metabolism as a determinant that enables leukemia and lymphoma cells to thrive, proliferate, and disseminate and (ii) discuss the importance of lipoproteins in shaping the tumor cell microenvironment, in the context of hematological malignancies.

## 2. Overview of Lipoprotein Metabolism

Lipoproteins are pluri-molecular complexes that serve as the principal carriers of lipids within the blood, lymph and interstitial liquids. By enabling lipid solubilization and transport in aqueous environments, lipoproteins play a central role in whole-body lipid homeostasis. They mediate the distribution of dietary lipids from the intestine to the liver, muscle, and adipose tissue; the delivery of hepatic lipids to peripheral tissues; and the recycling of excess cholesterol through reverse cholesterol transport. Lipoproteins are synthesized primarily by the liver and the intestine and share conserved structural organization. Each particle consists of a hydrophobic core enriched in cholesteryl esters (CEs) and triacylglycerols (TGs), surrounded by a polar phospholipid coat that contains apolipoproteins [34]. These apolipoproteins are not merely structural components but actively govern lipoprotein stability, trafficking, receptor recognition, and cellular uptake [35]. Based on particle size, density, lipid composition, and apolipoprotein content, plasma lipoproteins are classically divided into five major classes: chylomicrons, very-low-density lipoproteins (VLDLs), intermediate-density lipoproteins (IDLs), low-density lipoproteins (LDLs), high-density lipoproteins (HDLs), and lipoprotein(a) Lp(a) [34].

**Triglyceride-rich lipoproteins**—Chylomicrons are the largest triglyceride-rich particles produced by the enterocytes from dietary lipids, specifically cholesterol and fatty acids. Following secretion into the lymphatic circulation and subsequent entry into the circulation, chylomicron-derived triglycerides are hydrolyzed by lipoprotein lipase (LPL), allowing for lipid uptake by adipose tissue and skeletal muscle [36]. The resulting chylomicron remnants are then cleared by the liver through receptor-mediated endocytosis and further metabolized [37]. In parallel, the liver produces VLDL particles from both dietary-derived and endogenously synthesized lipids, which are secreted directly into the bloodstream. The progressive hydrolysis of TGs by LPLs, hepatic lipase (HL), and endothelial lipase (EL) generates smaller particles known as intermediate-density lipoproteins (IDLs) [38].

**Cholesterol delivery to peripheral tissues**—The further hydrolysis of IDLs by hepatic lipase (HL) results in the formation of LDLs, which are enriched in cholesterol and contain apolipoprotein B100 (ApoB100) as their primary structural proteins. LDLs are the major carriers of cholesterol in the body and are responsible for supplying cholesterol in tissues expressing the LDL receptor (LDLR) through endocytosis [39]. In the 1960s, lipoprotein(a) (Lp(a)) was discovered. Lp(a) is a lipoprotein similar to LDL in terms of lipid and lipoprotein composition, as it is attached to apolipoprotein-a. Despite extensive studies, the physiological role of Lp(a) and the mechanisms governing its clearance from the circulation remain incompletely understood [40]. Both LDL and Lp(a) metabolism have been extensively studied due to their strong association with cardiovascular disease risk [41].

**HDLs and reverse cholesterol transport**—High-density lipoproteins are the smallest and most dense lipoproteins and are synthesized by both the intestine and the liver [42]. Nascent HDL particles, which primarily contain apolipoprotein A-I and phospholipids, acquire unesterified cholesterol and additional phospholipids from peripheral cells. Through the process of reverse cholesterol transport, HDLs mediate the removal of cholesterol from peripheral cells and delivers it to the liver either directly, by binding to hepatic scavenger receptor B type 1 (SR-B1 receptor), or indirectly by transferring cholesterol to ApoB-rich lipoproteins that are subsequently cleared by the liver [43].

**Lipoprotein heterogeneity and oxidative modification**—Beyond this classical organization, it is now well recognized that each lipoprotein class comprises multiple subclasses that differ in size, density, lipid composition, and biological function. Distinct VLDL, LDL and HDL subclasses have been identified using ultracentrifugation and electrophoretic approaches, underscoring the functional diversity of circulating lipoproteins [44]. In addition, lipoproteins are susceptible to oxidative modification in the circulation. Oxidized LDLs (oxLDLs) are recognized by scavenger receptors such as CD36, and elevated levels of oxidized lipoproteins have been associated with cardiovascular diseases, type 2 mellitus diabetes, and various cancers [45].

Alterations in circulating lipoprotein levels are classically associated with metabolic disorders, including obesity, familial hypercholesterolemia, and atherosclerosis, and with cardiovascular disorders [46]. Dyslipidemia is also frequently observed as a secondary consequence of chronic conditions such as diabetes, renal dysfunction, and liver disease [47,48,49]. Increasing evidence now indicates that abnormal plasma lipoprotein profiles are a common feature of cancer and may actively contribute to tumor progression. In particular, lower levels of serum LDLs and HDLs and increased oxLDL levels have been consistently associated with disease progression across multiple solid tumors, including lung, prostate and breast cancer [50,51]. The mechanisms responsible for this association are not fully understood, but they might at least be partly explained by an increase in lipoprotein uptake and by elevated lipoprotein receptor levels on cancer cells [50]. A similar systemic rewiring of blood lipoproteins during tumor development has been observed in the context of hematological neoplasms. The following section will provide an overview of changes in blood lipoprotein levels during leukemic progression and address the main cellular mechanisms by which blood cancer cells can use lipoproteins to survive.

## 3. Importance of Lipoproteins in Hematological Malignancies

**Blood lipoprotein levels and blood cancer risk**—Leukemias are hematological cancers characterized by the uncontrolled proliferation of abnormal leukocytes. They are classified according to their cellular origin (myeloid or lymphoid) and disease kinetics (acute or chronic) [52]. Several clinical studies have explored the relationship between lipoprotein profiles and leukemia progression. In a retrospective case–control study on a Canadian cohort including 2124 CLL patients and 7935 control subjects, Mozessohn et al. (2016) studied the relationship between CLL diagnosis and dyslipidemia. They reported a higher prevalence of dyslipidemia preceding the diagnosis of CLL and an improved overall survival upon antidyslipidemic medication [53]. Consistently, in another single-center, retrospective study, the incidence of hypercholesterolemia was evaluated in 231 patients admitted for CLL. A high incidence of hypercholesterolemia was found for 75% of the CLL patients, and statin prescription upon diagnosis delayed the time to first treatment [27]. Further, serum metabolites were examined in a cohort of 29 untreated asymptomatic CLL patients and 9 control subjects using ^1^H nuclear magnetic resonance (NMR)-based metabolomics. High levels of VLDLs were found in patients at high risk of disease exhibiting unmutated IGHV (IGHV-UM) compared to IGHV-mutated patients (IGHV-M). This supports the idea that an increase in hepatic lipoprotein secretion cannot be totally excluded, especially since LDLs are derived from VLDLs [54]. However, the conclusions of the previous studies should be treated with caution due to the nature of the studies (single-center and retrospective). Furthermore, the first two studies suffer from potential bias. In fact, 40% of patients diagnosed with CLL were excluded from the first study due to the risk of missing monitoring data, and the second had a high proportion of patients treated with statin.

However, contrasting results have been reported in other studies, conducted with smaller cohorts of patients, which revealed reduced LDL and HDL levels in CLL patients compared to control patients at the time of diagnosis [31,55,56]. In addition, decreased total cholesterol (TC), LDL-C and HDL-C levels were associated with shorter time to first treatment and reduced survival and were proposed as prognosis markers [55]. Similar reduced total cholesterol and HDL levels were observed in acute lymphoblastic leukemia (ALL) [57,58], AML [59], and CML [60]. The reasons for the discrepancies in the conclusions of these studies compared to previous ones are difficult to assess due to the multiple biases that can be introduced in this type of study, such as differences in the time of diagnosis or patient ages. Further, blood LDL levels can be modulated by at least two phenomena: first, changes in the hepatic secretion of lipoproteins and second, variation in uptake by peripheral cells, including cancer cells. In AML and CLL, an inverse correlation has been observed between plasma LDL cholesterol and lipoprotein uptake by cancer cells, suggesting that one possible explanation for the reduced circulating LDLs at the time of diagnosis or during the course of leukemia is increased uptake by cancer cells [31,61]. In lymphoma, TC, LDL and HDL levels are also frequently reduced several years prior to diagnosis [28,62] and are associated with unfavorable outcomes [63]. Reduced HDL levels are also correlated with an increased risk of lymphoma [64] and MM [65].

In conclusion, the majority of data positions blood lipoprotein levels, particularly low LDL and HDL levels, as predictive markers of cancer progression in leukemia and lymphoma. However, there is a clear need for prospective studies to clarify the relationship between blood lipoprotein levels, their uptake by cancer cells and hepatic lipoprotein secretion in leukemia and lymphoma patients.

**Lipoprotein levels as a prognostic marker**—The prognostic value of plasma lipoprotein levels during the clinical course of leukemia and lymphoma is supported by substantial evidence. In a study conducted in 85 AML patients, cancer remission was associated with increased cholesterol levels [65]. Similar findings were reported in a study conducted in 64 ALL patients, where low levels of high-density lipoprotein cholesterol, elevated triglycerides, and elevated low-density lipoprotein cholesterol returned to normal values during remission [66]. Moreover, the level of Lp(a), which was found to be elevated at the time of diagnosis, also decreased after successful chemotherapy [59]. The same observation was reported in CLL, in which HDL and LDL levels increased only in patients achieving complete or partial remission post-chemotherapy [55]. Similarly, in ALL, total circulating cholesterol and HDL levels were only significantly increased after chemotherapy induction in patients with complete remission [57,67]. The same potential use of lipoprotein levels as a prognostic marker was suggested in lymphoma [68,69,70]. These data highlight the prognostic value of lipoproteins in leukemia and lymphoma.

### Importance of Lipoprotein Uptake for Blood Cancer Cells

**Figure 1:**
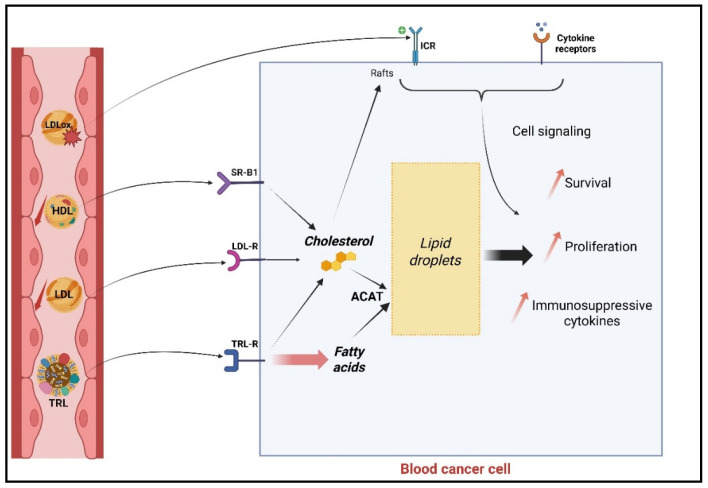
**Cellular mechanism linking circulating lipoproteins and blood cancer cell survival and proliferation.** In leukemia and lymphoma, malignant cells exhibit enhanced lipoprotein uptake capacity and contribute to lower circulating LDL and HDL levels. LDL particles are internalized through the LDL receptor (LDLR) and possibly other members of the LDLR family, leading to increased intracellular cholesterol levels and storage as cholesteryl esters (CEs) in lipid droplets. HDL-derived cholesterol esters are selectively transferred via scavenger receptor B type 1 (SR-B1) into cells. Through the formation of lipid rafts, cholesterol favors pro-survival and proliferative signaling pathways and the production of immunosuppressive cytokines. In addition, oxidized LDL (oxLDL) can be recognized by scavenger receptors such as cluster of differentiation 36 (CD36) or specific immune cell receptors (ICRs) such as the B-cell receptor (BCR) on B lymphocyte cells, activating pro-survival pathways. Triglyceride-rich lipoproteins (TRLs) are hydrolyzed by lipoprotein lipase (LPL) and undergo uptake by interacting with TRL receptors (TRL-R), releasing free fatty acids that can be taken up and used for oxidative metabolism and membrane synthesis. Collectively, increased lipoprotein uptake and lipid utilization support leukemic and lymphomatous cell survival, proliferation, metabolic adaptation, and resistance to therapy. Created in BioRender (2026). https://BioRender.com/1yct5sa.

*Role of LDL uptake*—LDLs are the major cholesterol transporters in the circulation. Early studies suggested that cancer cells from AML and CML patients during blast crisis have a higher capacity than healthy and ALL cells to degrade radiolabeled ^125^I-LDL through a receptor-mediated process [29,71]. LDL uptake by cancer cells was particularly studied in AML. In fact, LDL uptake was inversely correlated with blood cholesterol levels, suggesting that high LDL uptake by cancer cells may contribute to hypocholesterolemia [61]. Further, LDLR expression levels were inversely correlated with patients’ overall survival [72]. In vitro, LDL uptake through LDL receptors was shown to confer resistance to chemotherapy [73], and the LDL-mediated delivery of chemotherapeutics was proposed as a promising approach [74]. However, besides LDLR, several reports suggested that other lipoprotein receptors such as lipoprotein receptor-related proteins (LRPs) may be involved in LDL uptake [75]; a clear investigation of the relative contribution in LDL uptake of the various family receptors is still needed (Figure 1).

The importance of LDL uptake was particularly investigated in CLL. Notably, CLL cells have a higher LDL uptake and LDLR expression level compared to B cells from healthy individuals [56]. Despite elevated LDLR levels, a lower LDL degradation rate was reported in CLL [56,76]. The reasons for this lower LDLR activity are unclear but might be due to several factors, including the quiescent state of circulating CLL cells [77]. Indeed, the signal transducer and activator of transcription (STAT3) signaling pathway is constitutively activated and crucial for CLL cell growth and proliferation [78]. In vitro studies showed that LDL uptake by CLL cells increases cell proliferation through a higher STAT3 phosphorylation rate This result was only observed upon the stimulation of the cells with IL-2 and TLR-7 agonists and absent in the unstimulated quiescent state. One study also revealed that this effect was mediated by an increased cholesterol content [79]. Cholesterol content seems to be an important driver of CLL cell proliferation. Many studies compared cholesterol content between healthy and cancer B cells and found opposite results. Indeed, several studies reported higher cholesterol content in CLL cells [79,80], while others reported lower levels [81,82]. The origin of these conflicting results are not clear but may be due to the purification procedures of the cells or the lipid extraction procedures, and further clarifications are needed. It seems that in CLL cells, the ratio between free and esterified cholesterol is low, in line with a higher cholesterol store. In addition, the treatment of leukemic cells from CLL and ALL patients with inhibitors of cholesterol esterification caused a sharp decline in cell proliferation, supporting the crucial role of cholesterol storage [31]. Finally, in CLL, continuous B-cell receptor (BCR) stimulation is believed to promote the growth and survival of leukemic cells through the activation of multiple signaling pathways. Among others, oxLDL, frequently found in the blood circulation of cancer patients [83], was shown to bind to BCR on CLL cells, but it did not provoke cell signaling or cell proliferation [84,85]. The exact functional consequence of this interaction remains unclear but requires further investigation, particularly in proliferative niches (Figure 1).

A crucial role for LDL uptake and increased LDLR expression was also described in Burkitt’s lymphoma cell proliferation [86] and in MM survival through the inhibition of ferroptosis [87]. Likewise, the cytoplasm of high-grade lymphoma cells often contains lipid vacuoles. Treatment with acyl-coenzyme A: cholesterol acyltransferase-1 (ACAT) inhibitors led to cell apoptosis mediated by increased endoplasmic reticulum stress [88], supporting the importance of intracellular cholesterol content.

*Scavenger receptor B type 1 (SR-B1) and HDL uptake*—SR-B1 is a cell membrane HDL receptor involved in bidirectional cholesterol ester uptake. Transfer from HDLs and plasma involves a non-endocytic pathway [89]. As previously discussed, low circulating HDL levels were frequently observed in leukemia and lymphoma and might reflect high SR-B1 activity [55,57,59,60,62]. This was suggested by the fact that, unlike normal B cells, CLL cells express high levels of SR-B1 (Figure 1). Moreover, low cholesterol synthetic HDL nanoparticles induce the apoptosis of CLL cells, suggesting that cholesterol from HDLs promotes the survival of CLL cells and that targeting this pathway is a therapeutic option [90]. This pathway is also important in high-grade lymphomas since SR-B1 inhibitors induce cell death and exhibit a synergistic effect with ACAT inhibitors [91].

*Triglyceride-rich lipoprotein (TRL) usage by cancer cells*—Lipoprotein lipase (LPL) plays a major role in lipoprotein metabolism by catalyzing the hydrolysis of triglycerides contained in TRLs like chylomicrons and VLDLs, thereafter releasing free fatty acids that can be absorbed by the cells [92]. Unlike normal lymphocytes, CLL cells do express LPL [93], with its expression level being particularly high in patients with shorter treatment-free survival and with a trend for shorter median overall survival [94,95,96]. The importance of LPL for CLL cell survival is also supported by the fact that the treatment of CLL cells with orlistat (LPL inhibitor) is cytotoxic for CLL cells, an effect enhanced by simultaneous incubation with fludarabine [97]. However, it was reported that orlistat can also inhibit fatty acid synthase (FASN), a crucial enzyme involved in the endogenous production of fatty acids. So, reduced fatty acid synthesis can explain in part the effects observed in this study [98]. Other studies have suggested that LPL is catalytically inactive in CLL cells [99]. Non-catalytic roles have been attributed to LPL, including the facilitation of lipoprotein endocytosis by bridging TRLs and LDLs to cell surface heparan sulfate proteoglycans (HSPGs) [100]. Other studies have suggested that LPL may also mediate cell–cell interactions between CLL cells and the cells of the tumor microenvironment (TME). Although these mechanisms have not been clearly demonstrated in CLL, they warrant further investigation, as they may contribute to a better understanding of the survival advantage observed in cells with high LPL expression levels.

In conclusion, clinical, epidemiological and biological reports highlight a strong association between dysregulated lipoprotein metabolism and hematological malignancies at risk of progression. Despite important breakthroughs over the past few decades, the exact mechanisms underlying this correlation remain to be explored. A deeper mechanistic understanding of how lipoprotein trafficking and usage shape leukemic biology may uncover novel biomarkers and therapeutic vulnerabilities. Furthermore, whether lipoprotein abnormalities are a cause and/or a consequence of cancer remains to be established.

## 4. Does Lipoprotein Metabolism Shape the Tumor Microenvironment in Hematological Malignancies?

The tumor microenvironment (TME) plays a crucial role in the initiation, progression, and therapeutic resistance of hematological malignancies. Within anatomical niches, such as bone marrow, and secondary lymphoid organs, leukemic cells interact with a complex network of immune and stromal populations that collectively establish a leukemia-supportive ecosystem. These cellular components include T lymphocytes, monocytes/macrophages, dendritic cells, bone marrow adipocytes, natural killer (NK) cells, mesenchymal stromal cells, and endothelial and fibroblastic populations. Through both direct cell–cell contacts and the secretion of cytokines, chemokines, growth factors, and metabolites, the TME promotes tumor cell survival, proliferation, immune evasion, and resistance to therapy [101]. A deeper understanding of cancer cell–TME interactions is therefore essential to identify novel therapeutic vulnerabilities and to overcome treatment resistance [102]. While the contribution of glucose and amino acid metabolisms to the hematological TME has been extensively studied, the role of lipids and associated lipoprotein metabolism remains underexplored. The aim of this section is to outline the principal mechanisms by which lipoprotein metabolism may contribute to shaping the tumor microenvironment in blood cancers.

**The tumor microenvironment control of lipoproteins is a target of malignant cells**—The enhanced uptake and use of low-density lipoproteins (LDLs) have been reported in several hematological malignancies, including leukemia. Importantly, lipoprotein metabolism appears to be tightly regulated by microenvironmental cues rather than being solely tumor cell-intrinsic [61,71]. Indeed, in CLL, malignant B cells proliferate primarily within specialized lymphoid structures, so-called proliferation centers, where they receive activating signals from the B-cell receptor (BCR), toll-like receptors (TLRs), tumor necrosis factor (TNF) family ligands, cytokines, and chemokines [101]. In this context, McCaws and colleagues demonstrated that in vitro, LDL activates pro-proliferative signaling pathways in primary CLL cells only when cells are co-stimulated with IL-2 and TLR-7 agonists, mimicking T- and dendritic cell-derived signals within the niche [79]. Moreover, the engagement of cluster of differentiation 40 (CD40) and cytokine stimulation upregulates the expression of multiple lipoprotein receptors including LDLR, VLDLR, and LRP-1,5,6,8, although the functional relevance of several of these receptors remains incompletely understood [103]. However, since several of these receptors have a key role in the supply of cholesterol and triglycerides—two crucial metabolites for cell survival and proliferation—it is conceivable that the effects of CD40 and cytokines on the survival and proliferation of cancer cells are mediated, at least in part, through lipoprotein receptors. In AML, LDL uptake is also enhanced by cytokine signaling through autocrine and paracrine mechanisms, involving potentially IL-1β and TNF-α [104]. Together, these observations support a bidirectional regulatory loop where microenvironmental inflammatory and immune signals modulate lipoprotein uptake by tumor cells, which in turn may reinforce malignant growth and metabolic adaptation.

**T lymphocytes, lipoprotein metabolism and antitumor immunity**—T lymphocytes play a crucial role in antitumor immune surveys [105]. However, in several hematological malignancies, including CLL, T cells display defects, among which exhaustion, senescence, impaired synapse formation, and reduced cytotoxic activity are all correlated to poor clinical outcomes [106]. Recent evidence indicates that altered lipid handling directly contributes to T-cell dysfunction. T cells isolated from CLL patients display reduced LDLR expression and the impaired uptake of exogenous cholesterol, leading to defective proliferation and compromised immunological synapse [107]. Also, as cancer cells have a high LDL uptake capacity, competition between lipoproteins may explain the cholesterol deficiency observed in neoplastic T lymphocytes. These elements suggest that dysregulated lipoprotein availability or use within the TME may suppress antitumor immunity.

***C*D36 and lipoprotein-derived lipids in leukemia progression and immune suppression**—CD36 is a scavenger receptor expressed in many immune and non-immune cells and serves as both a signaling receptor and a transporter of long-chain free fatty acids [108]. In AML, CD36-mediated lipid uptake promotes leukemic cell survival and metabolic fitness [109]. Apolipoprotein C2 (ApoC2), a component of chylomicrons, VLDLs, and HDLs, is highly expressed in aggressive forms of AML blast in comparison to normal bone marrow cells. In AML, ApoC2 interacts with CD36 to activate LYN and extracellular signal-regulated kinase (ERK) signaling, enhancing proliferation [17]. Beyond supporting tumor metabolism, CD36 also contributes to immune evasion. AML cells can sense oxidized LDLs through CD36, and in synergy with palmitate uptake, they can activate immunosuppressive gene programs that inhibit T-cell responses and promote resistance to hypomethylating agents [110]. These findings highlight CD36 as a critical metabolic–immunological nexus linking lipid availability to tumor aggressiveness and immune suppression.

**Macrophages and oxidized lipoproteins in the bone marrow niche**—Tumor-associated macrophages (TAMs) are abundant components of the leukemia and lymphoma microenvironment and exhibit remarkable functional plasticity [111]. In MM, macrophages accumulate in the bone marrow and promote tumor growth, angiogenesis, and immune suppression through cytokine secretion, extracellular vesicle release, and direct cell–cell interactions [112]. oxLDL, long recognized as a driver of foam cell formation in atherosclerosis, has recently been detected in the bone marrow microenvironment of MM patients [113]. In bone marrow, oxLDL may originate from the circulation and also from myeloperoxidase activity in monocytes and granulocytes. It was observed that in bone marrow, oxLDLs limit the toxicity of bortezomib and ixazomib, two proteasome inhibitors, on MM cells. These observations suggest that the oxidative modification of lipoproteins within the tumor niche may reprogram macrophage function and contribute to therapeutic resistance [114].

**Bone marrow adipocytes as lipid reservoirs**—Bone marrow adipocytes (BMAs) represent a substantial fraction of the marrow compartment and exhibit a distinct metabolic identity in comparison to white adipocyte characterized by enrichment in genes encoding for cholesterol metabolism and lipoprotein transport pathways [115]. BMAs directly support the growth, survival, and dissemination of multiple myeloma and AML cells, through the supply of essential metabolites like fatty acids and glutamine and adipokine secretion [116,117,118]. Notably, increased marrow adiposity has been associated with pathologic conditions like obesity and aging, as well as reduced blood-circulating HDL cholesterol [119,120]. As discussed below, low HDL levels are frequently observed during the course of hematological malignancies; this raises the intriguing possibility that systemic dyslipidemia may indirectly remodel the bone marrow niche toward a tumor-permissive state. However, the mechanistic links between HDL metabolism, BMA biology, and leukemia progression remain poorly understood.

**Mesenchymal stromal cells and local lipoprotein production**—Bone marrow mesenchymal stromal cells (BM-MSCs) are multipotent progenitors that contribute to hematopoietic support, extracellular matrix remodeling, and immune regulation [121]. Single-cell transcriptomic analyses of CLL and AML bone marrow have revealed that subsets of MSC could secrete apolipoprotein E (ApoE) [103,122]. ApoE is an abundant circulating protein involved in the transport of triglycerides. In CLL, ApoE was shown to be toxic to proliferating CLL B cells by inducing cell death through ferroptosis [103], to a lesser extent in progressive CLL and Richter transformed cells. In AML, an inverse association was suggested, since ApoE from MSC favors cell growth probably through MAPK signaling [123]. However, various ApoE isoforms are described, and the function of each isoform should be evaluated. Also, ApoE has various receptors that are associated with the signaling of multiple cells, and mechanistic studies are required to clarify which receptors are responsible for the observed effects.

**Figure 2:**
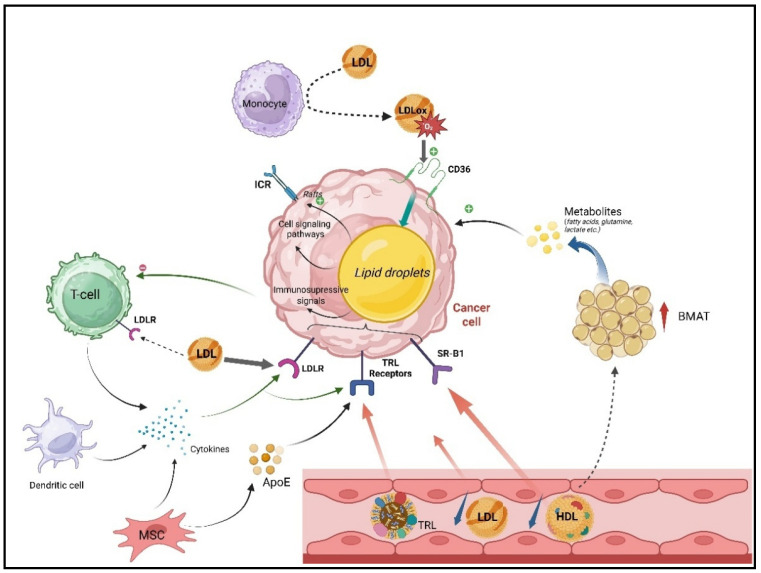
**Proposed model explaining the role of lipoprotein metabolism in shaping the TME in hematological malignancies.** Increased low-density lipoprotein (LDL) uptake by malignant cells enhances intracellular cholesterol content, promoting cell survival and proliferation and supporting lipid raft formation, thereby strengthening pro-survival signaling through immune cell receptors (ICRs), such as the B-cell receptor (BCR). LDL also induces the metabolic reprogramming of cancer cells, leading to the secretion of immunosuppressive cytokines that impair T-cell function. Increased LDL uptake by cancer cells reduces LDL availability within the tumor microenvironment (TME) and contributes to T-cell dysfunction. In the TME, LDL can be oxidized by myeloperoxidase released from monocytes and granulocytes. In acute myeloid leukemia (AML), oxidized LDL (oxLDL) promotes immunosuppression and chemotherapy resistance through a CD36-mediated pathway. Reduced circulating high-density lipoprotein (HDL) levels, frequently observed in hematological malignancies, may reflect increased uptake via the scavenger receptor (SR-B1). Low HDL levels are associated with increased bone marrow adipose tissue (BMAT), which augments the supply of various metabolites (fatty acids, cholesterol, and glutamine) to the cancer cell. Additionally, malignant cells express various receptors for triglyceride-rich lipoproteins (TRLs), enabling the binding of apolipoprotein E (ApoE)-rich particles, as well as ApoE derived from bone marrow stromal cells (BMSCs). Notably, in chronic lymphocytic leukemia (CLL), lipoprotein receptor expression is modulated by locally produced cytokines, potentially amplifying their functional activity. Created in BioRender (2026). https://BioRender.com/1yct5sa.

By integrating all these data, we propose the following hypothetical model to explain how lipoproteins can favor the establishment of a tumor permissive microenvironment in hematological malignancies. First, LDL uptake by cancer cells increases intracellular cholesterol stores, promoting cell survival and proliferation. Lipid raft formation potentiates pro-survival cell signaling through specific immune cell receptors (ICRs), like B-cell receptor or T-cell receptor signaling. LDL also reprograms cancer cells and leads to the secretion of immune-suppressive cytokines, as well as reduced LDL availability in the TME, which leads to T-cell dysfunction. Furthermore, in the TME, LDLs are prone to oxidation via the myeloperoxidase activity of monocytes and granulocytes. In AML, oxLDLs are involved in immunosuppression and chemotherapy resistance through a CD36-mediated pathway. In addition, low levels of circulating HDLs are frequently observed during hematological malignancies and can be a consequence of increased uptake mediated by the SR-B1 receptor. Low circulating HDL levels are also associated with an increase in bone marrow adiposity, increasing the supply in various metabolites like fatty acids, cholesterol and glutamine. Finally, cancer cells express various TRL receptors that can bind to lipoprotein-rich ApoE (i.e., chylomicrons, VLDLs and IDLs) and also free circulating ApoE or that derived from BMSCs. Finally, particularly in CLL, the expression of lipoprotein receptors is shown to be sensitive to cytokines produced locally, with a possible potentiation of their function (Figure 2).

## 5. Perspectives and Open Questions

The continuous invasion and rapid growth of tumor cells require a substantial energy supply. Metabolic reprogramming is commonly used to meet the material and energy requirements of tumor cells. Malignant cells are supported by survival and growth factors provided by populations of various cell types within the proliferative niches of lymph nodes, bone marrow, and secondary lymphoid organs. Still, there is a need to better understand how the TME supports cancer cells in order to uncover new cancer cell vulnerabilities from a therapeutic perspective. In this review, we discussed the importance of considering lipoprotein metabolism during blood cancer progression.

In hematological malignancies, lipoprotein metabolism seems to support cancer cell function via at least two mechanisms. First, lipoproteins provide substantial amounts of metabolites that sustain cancer cell survival and proliferation. Indeed, increasing evidence reports that abnormal blood lipoprotein profiles, particularly low LDL and HDL levels, are frequently observed during leukemia and lymphoma progression. One current hypothesis is that the increased LDL uptake of cancer cells is responsible for blood starvation, given that in several studies, blood LDL reduction correlates with cancer cell LDLR activity. However, we cannot exclude the fact that liver lipoprotein secretion is impaired during cancer progression. Future investigations should also clarify the contribution of lipoprotein uptake by cancer cells and by other immune cells. Several therapeutic strategies have been developed to target the high lipoprotein uptake capacity of blood cancer cells. For instance, the in vitro efficacy of chemotherapy incorporation in LDL was demonstrated in several pathologies including AML [74]. Similarly, reconstituted lipid-poor HDLs were broadly investigated for their use in many cancers, including blood cancers [90]. The identification of the precise mechanisms underlying the uptake of lipoproteins by cancer cells, as well as the pattern of lipoprotein receptors expressed by cancer cells during cancer progression, and their relative contribution to lipoprotein uptake is needed. This can allow for the identification of new therapeutic strategies. Also, in recent years, light was shed on the characterization of various lipoprotein subclasses and oxidized forms, as well as their functional differences in pathophysiological contexts. There is still a need to evaluate whether there are qualitative changes in blood lipoproteins (VLDL, LDL, HDL subclasses) in hematological malignancies and what their respective roles are in cancer cell function.

The second mechanism by which lipoprotein metabolism may support the progression of hematological malignancies is through the shaping of the tumor microenvironment. While this aspect has been extensively studied in solid tumors, relatively few studies have addressed it in the context of blood cancers. T cells plays a crucial role in antitumor immunity, and cholesterol is essential for T-cell activation and immunological synapse formation. In CLL, T cells display functional impairments that have been linked to the defective uptake of exogenous cholesterol [107]. This cholesterol deficiency may result from competition between T cells and cancer cells for LDL uptake within the TME. However, the differential uptake capacity of the various cell populations comprising the TME has not been clearly assessed. Moreover, the precise mechanisms governing lipoprotein uptake in these populations remain poorly characterized. Identifying preferential lipoprotein uptake mechanisms in cancer cells could therefore reveal a novel therapeutic target. Beyond the well-described lipoprotein uptake mediated by lipoprotein receptors, alternative mechanisms may contribute to this process. Notably, heparan sulfate proteoglycans (HSPGs) act as TRL receptors in the liver and facilitate lipoprotein presentation to specific receptors [124]. Among them, syndecan-1 is highly expressed on the surface of MM cells and mediates interactions with the components of the TME. However, its potential role in lipoprotein uptake has not yet been investigated. More broadly, the contribution of HSPGs to cancer cell interactions with the components of the TME and the contribution of lipoproteins or apolipoproteins in these processes remain largely unexplored and may uncover new vulnerabilities of cancer cells. Finally, macropinocytosis has been described to support cancer cell metabolism through the uptake of extracellular materials. Its specific contribution to lipoprotein internalization has not been studied so far and may provide new therapeutic perspectives in the field.

## Data Availability

No new data were created or analyzed in this study.

## Abbreviations

The following abbreviations are used in this manuscript:

ACAT	acyl-coenzyme A: cholesterol acyltransferase-1
ALL	acute lymphoblastic leukemia
AML	acute myeloid leukemia
ApoA	apolipoprotein A
ApoB100	apolipoprotein B100
ApoC2	apolipoprotein C2
ApoE	apolipoprotein E
BMA	bone marrow adipocyte
BCR	B-cell receptor
BM-MSC	bone marrow mesenchymal stromal cells
CD36	cluster of differentiation 36
CD40	cluster of differentiation 40
CE	cholesteryl ester
CLL	chronic lymphocytic leukemia
CML	chronic myelogenous leukemia
DLBCL	diffuse large B-cell lymphoma
EL	endothelial lipase
ERK	extracellular signal-regulated kinase
FA	fatty acid
FABPs	fatty acid-binding proteins
FASN	fatty acid synthase
FATPs	fatty acid transport proteins
HDL	high-density lipoprotein
HL	hepatic lipase
HSPGs	heparan sulfate proteoglycans
ICRs	immune cell receptors
IDLs	intermediate-density lipoproteins
IGHV-UM	immunoglobulin heavy chain variable region-unmutated
IGHV-M	immunoglobulin heavy chain variable region-mutated
LDLR	low-density lipoprotein receptor
Lp(a)	lipoprotein(a)
LPL	lipoprotein lipase
LRPs	lipoprotein receptor-related proteins
MAPKs	mitogen-activated protein kinases
MCL	mantle cell lymphoma
MM	multiple myeloma
NK	natural killer
NMR	nuclear magnetic resonance
oxLDL	oxidized LDL
SLC27	solute carrier family 27
SR-B1	scavenger receptor B type 1
STAT3	signal transducer and activator of transcription
TAM	tumor-associated macrophage
TG	triacylglycerol
TME	tumor microenvironment
TNF	tumor necrosis factor
